# Comparative Study of Recessive Spherical Headed Silicone Intubation and Endonasal Dacryocystorhinostomy under Nasal Endoscopy for Nasolacrimal Duct Obstruction

**DOI:** 10.1038/s41598-017-07293-7

**Published:** 2017-08-10

**Authors:** Hui-yi Deng, Tao Wang, Xue-kun Huang, Qin-tai Yang, Shi-qi Ling, Wei-hao Wang, Mei-jiao Li, Fang-qin Ning, Ge-hua Zhang

**Affiliations:** 1 Department of Otorhinolaryngology-Head and Neck Surgery, The Third Affiliated Hospital of Sun Yat-sen University, Guangzhou, Guangdong 510630 P.R. China; 2 Department of Ophthalmology, The Third Affiliated Hospital of Sun Yat-sen University, Guangzhou, Guangdong 510630 P.R. China; 3 Department of Medical Record Management, The Third Affiliated Hospital of Sun Yat-sen University, Guangzhou, Guangdong 510630 P.R. China

## Abstract

Between July 2014 and November 2015, we compared the curative effects and cost-effectiveness of two kinds of nasal endoscopic surgery for nasolacrimal duct obstruction (NLDO) in a single-centre, two-armed clinical trial with a 1-year follow-up. We included two groups: a recessive spherical headed silicone intubation (RSHSI) group and an endonasal dacryocystorhinostomy (En-DCR) group; both received nasal endoscopy. Patients were recruited from the Otorhinolaryngology and Ophthalmology departments. The main outcome measures were epiphora improvement (classified as cure, effective, or invalid), cost-effectiveness, visual analogue scale (VAS) intraoperative pain score, bleeding volume, operating time, hospitalisation time, total cost, and VAS postoperative epiphora score. No significant group difference was identified in postoperative epiphora VAS scores (P > 0.050) or success rate (P = 0.406). However, average VAS intraoperative pain score, operating time, bleeding volume, hospitalisation time and total cost in the RSHSI group were clearly lower to those in the En-DCR group (P = 0.000). In conclusion, RSHSI under nasal endoscopy can provide similar treatment outcomes to En-DCR. RSHSI has advantages including minimal invasiveness, reduced risk, shorter duration of surgery and hospitalisation, reduced intraoperative discomfort, and lower financial burden, which is more acceptable to patients. Thus, RSHSI may be the preferred option for NLDO.

## Introduction

Lacrimal duct obstruction is common among patients with epiphora and can affect quality of life. The guiding treatment principle for this condition is to restore or rebuild the lacrimal duct drainage channel. The classic operation is external dacryocystorhinostomy (Ex-DCR), which is an invasive surgical procedure requiring skin incision and osteotomy^1, 2^. With the development of endoscopy, endoscopic endonasal dacryocystorhinostomy (En-DCR) has become popular because of its minimal invasiveness, good visualisation, short operation and hospitalisation durations, limited bleeding, and high success rate. However, to a degree, the procedure is traumatic, in that it can sometimes disrupt the lacrimal pump system^3–8^.

**Figure 1:**
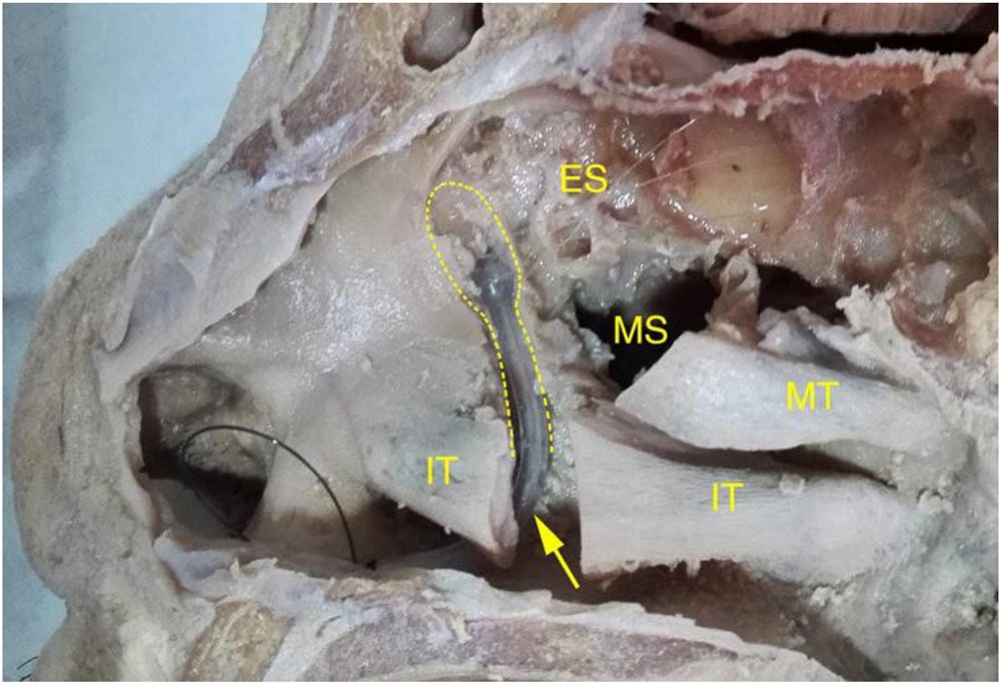
Pre-experiment RSHSI was initially performed on cadavers. The yellow dashed line represents the lacrimal sac and nasolacrymal duct. The yellow arrow represents the Spherical Headed Silicone Tube. IT, inferior turbinate; MT, middle turbinate; M, maxillary sinus; ES, ethmoid sinus.

**Figure 2:**
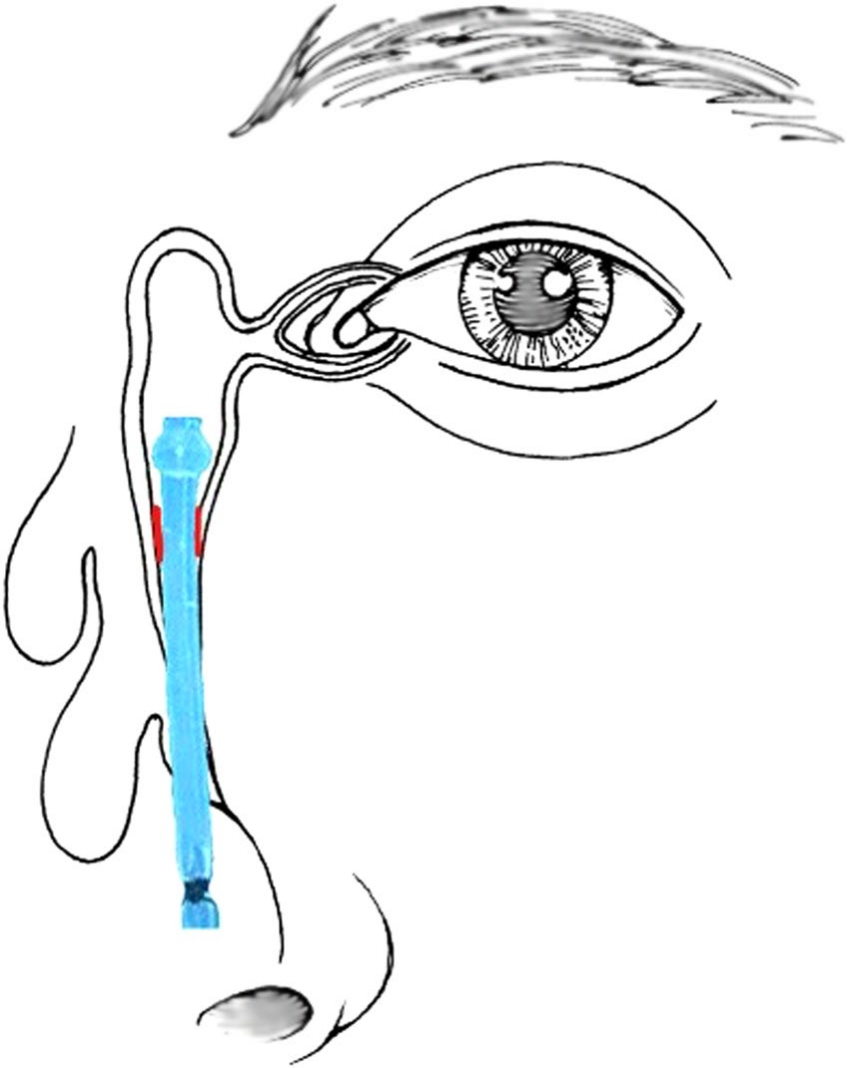
Recessive Spherical Headed Silicone Intubation under the endoscopy. The Red lines represent the obstruction in nasolacrimal duct. The Blue canaliculi represents the recessive spherical headed silicone tube (Drawn by Q.Y.).

Currently, many surgeons prefer to use recessive spherical headed silicone intubation (RSHSI) for the treatment of nasolacrimal duct obstruction (NLDO). RSHSI performed under nasal endoscopy is non-disruptive, in terms of the medial canthal ligament, and preserves the lacrimal pump system. Prior to any formal experiments, RSHSI was initially performed on cadavers to allow the procedure to be tested (Fig. 1 and note that we had obtained approval for use of cadavers in the clinical study and we had followed all the right procedures of the anatomists of Sun Yat-Sen University, the methods in this study was complied with the tenets of the Declaration of Helsinki). Dongguang Liu subsequently improved the technology and quality of RSHSI (Fig. 2), avoiding the degradation that occurs in traditional silicone tubes^9^; the efficiency was estimated to be above 90%. RSHSI offers considerable advantages, such as being minimally invasive, safe, cost-effective, amenable to performance on an outpatient basis, and able to avoid structural and functional damage to the lacrimal duct^5, 10^. Moreover, silica gel is non-toxic and non-irritating. When failure occurs, the same operation can be performed again or converted to En-DCR, especially for patients who require urgent intraocular surgery or are in poor condition^5, 11^. However, traditional surgery performed by ophthalmologists alone can result in numerous complications, such as nasal submucosa pseudocanal and nasal mucosa damage, scar formation, punctiform orifices, and haemorrhage. In this study—a joint project between otorhinolaryngologists and ophthalmologists—we investigated RSHSI performed under nasal endoscopy in terms of its intraoperative visualisation^1, 12–14^, lower risk of complications, and cost-effectiveness. We also sought to determine whether it can identify simultaneous ear, nose and throat (ENT) disorders, such as septoplasty^12^. In summary, we investigated the efficacy and cost-effectiveness of RSHSI.

## Methods

### Design overview

This two-armed trial examining treatment of NLDO was registered at http://www.ClinicalTrails.gov with the identifier NCT02636257 on September 12, 2015. All methods were performed in accordance with relevant guidelines and regulations, and all patients were asked to provide written, informed consent before enrollment.

### Sample size calculation

Calculation of the sample size for this trial was based on the rate of effectiveness, defined as 90% in both groups. For this calculation, we used the following parameters: type I error of 2.5%, 80% power, and a non-inferiority margin of 10%. Using these parameters, the sample size obtained was 141 per group. However, since this was only a 2-year program for a pilot study (conducted out of Sun Yat-sen University, China), we decided to use a smaller initial sample size (25 per group), with no interim analyses or stopping guidelines applied. Nevertheless, we followed the randomised controlled trial (RCT) rules carefully to allow better and further research in the future.

### Setting and participants

This comparative, randomised controlled trial was undertaken at the Third Affiliated Hospital of Sun Yat-sen University. The university ethics committee reviewed, approved, and monitored the study protocol. Between July 2014 and November 2015, patients suspected of NLDO were included when they met the following criteria: at least 18 years of age; able to withstand surgery; a sufficient level of education to understand the study procedures, communicate with site personnel and adhere to the follow-up; no lacrimal tumour or acute inflammation; NLDO on digital subtraction dacryocystography; clinical diagnosis of NLDO based on the presence of epiphora or purulent secretions; obstructed lacrimal irrigation with purulent reflux, such that the cannular probe could enter into the inferior meatus; and provision of informed consent, both verbally and in writing. Among these patients, those who met the following criteria were excluded: children; poor health; allergic to anaesthetics; nasolacrimal duct abnormalities; or prior failure of the same surgery. Patients enrolled into the study who were subsequently found to meet the following criteria were also excluded: postoperative infection and persistent inflammation, or artificial duct implantation failure during operation. All patients agreed to be randomly assigned to either the RSHSI or En-DCR group.

### Randomisation and masking

The randomisation procedure, carried out before participant enrollment, used a computer-generated random-number sequence to assign the patients to the two groups. The surgical team comprised one ENT surgeon (Q.Y.) and one ophthalmologist (T.W.). All patients and investigators were aware of the study group assignment, except for the data collector.

### Surgical approach

**Figure 3:**
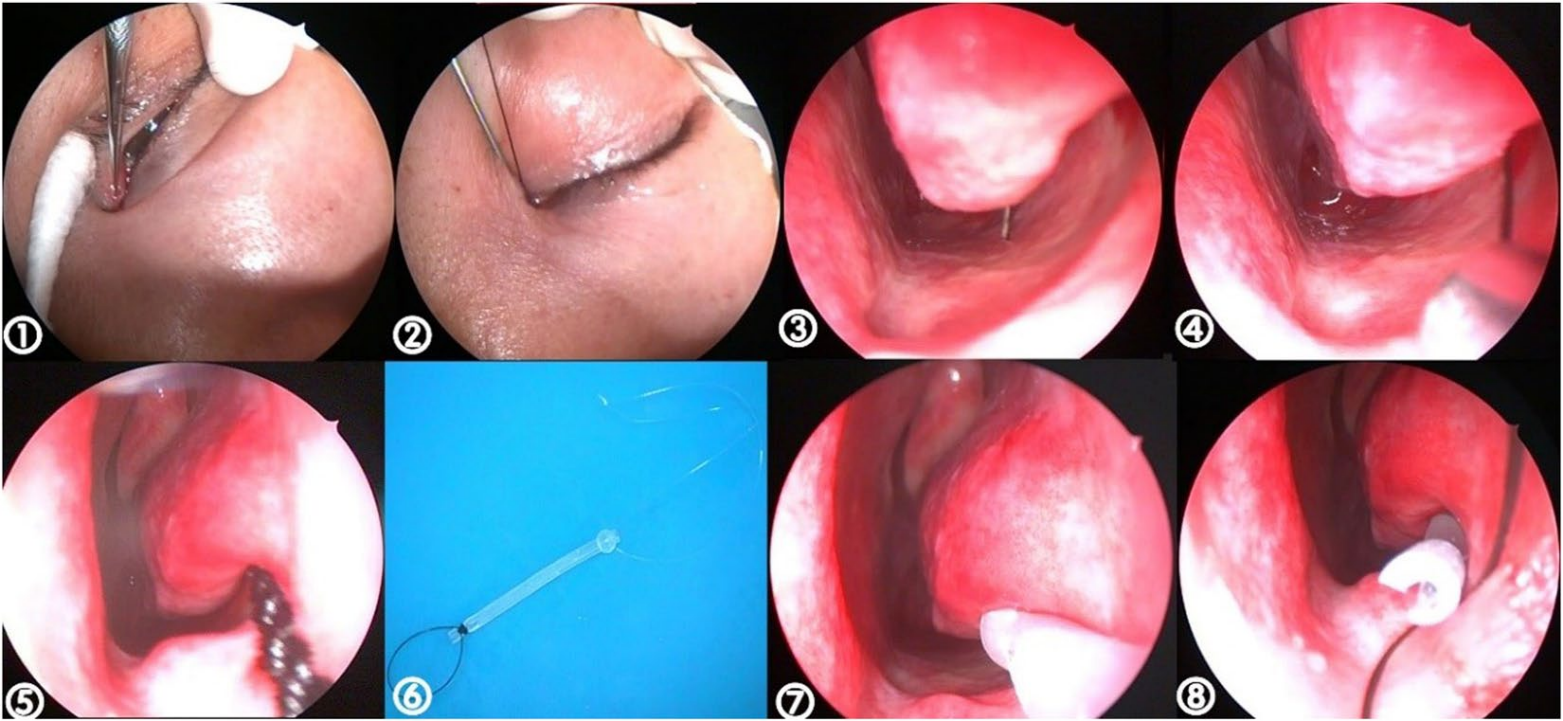
RSHSI performed under nasal endoscopy (①~⑧). No. ⑥ shows the spherical silicone tube (Guanghzhou BoShi health-care Institution: hollow circular, 3.5 cm long, 4.2 mm in diameter of spherical end, 2.5 mm in diameter of the duct, a 90 mm ± 10 mm circle line in the head, a 15 mm ± 5 mm long line in the tail, registration certificate number was 2260816).

**Figure 4:**
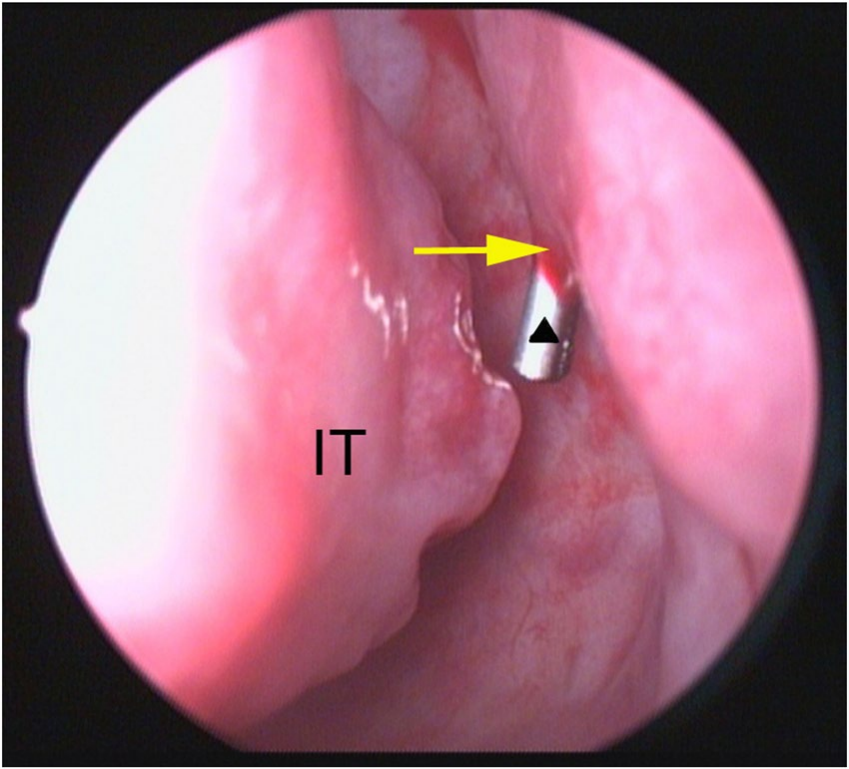
Under nasal endoscope, the wire-threaded hollow probe (black triangle) could be seen enter the inferior meatus through the opening of the nasolacrimal duct (yellow arrow). IT, inferior turbinate.

**Figure 5:**
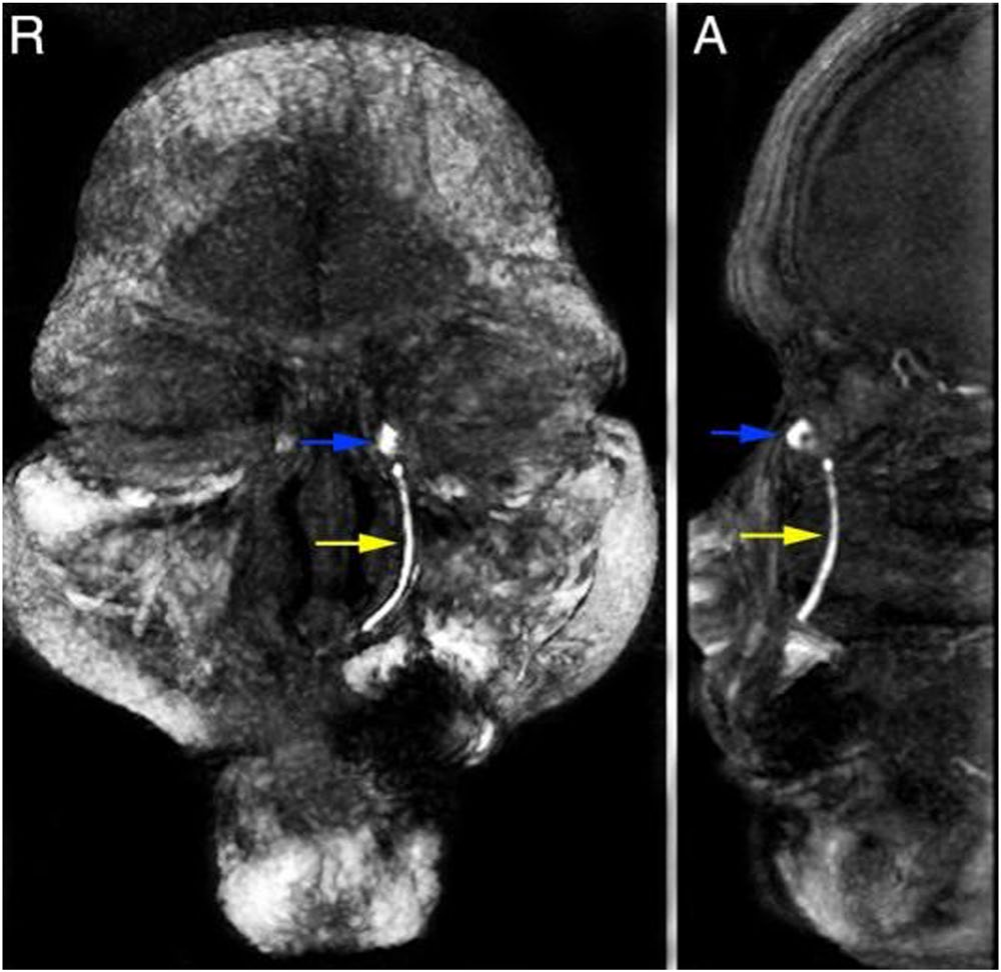
The MRI after RSHSI. The bule arrow represents the lacrimal sac. The yellow arrow marks the Spherical Headed Silicone Tube. R, Right; A, Ahead.

Recessive spherical headed silicone intubation performed under nasal endoscopy (Fig. 3): surgery, preferably performed under local anaesthesia, involved the regular use of disinfectants, sterile towels, and exposure of the operative side. As an anaesthetic, 2% lidocaine infiltration was applied to the inferior orbital nerves, lacrimal punctum, and lacrimal sac. The nasal cavity was packed with gauze soaked in 2% ephedrine, and operative eye surface anaesthesia, in the form of 1% tetracaine, was administered before the procedure began. A tear dilator was used to dilate the upper lacrimal point. Then, a wire-threaded hollow probe was inserted from the lacrimal punctum and lacrimal ductule into the lower end of the inferior meatus. Using a nasal endoscope, the probe could be seen to enter the inferior meatus through the opening of the nasolacrimal duct (Fig. 4). A line was then placed into the inferior meatus and 5 mL physiological saline was applied. The line was then removed and the probe extracted. The line and dilated cord were tied together to re-expand the canalis nasolacrimalis. Similarly, we inserted a spherical silicone tube from the nasolacrimal duct to the bulbous part of the lacrimal sac, hooked the abovementioned line, clipped the projecting part of the tube, and fixed it in place (Fig. 5). Unobstructed lacrimal irrigation was then performed to complete the operation (note: informed consent was obtained from the patient for the images publication).

Endonasal dacryocystorhinostomy: after general anaesthesia, the standard procedure began with instillation of vasoconstrictive and local anaesthetic agents. A 2.0 cm × 1.5 cm incision was made in the lateral nasal wall mucosa, including the periosteum anterior to the uncinate process, just below the attachment of the middle turbinate to the lateral nasal wall, keeping the mucosa between the middle turbinate and the lateral nasal wall intact. The mucosa was lifted and excised, then fixed to the olfactory cleft, exposing the maxilla frontal processes and the anterior lacrimal bone, and the bone was removed using a rongeur. A bony osteon of sufficient size, 1.0 cm × 1.5 cm, was formed to expose the lacrimal sac. The sac was excised to form U-shaped flaps that were glued to the front of the uncinate process. The initial lateral nasal wall mucosa was reset on the maxilla frontal processes. The lacrimal sac flaps were obturated with a gelatin sponge through the bony osteon, and the lacrimal sac cavity was obturated with a gelatin sponge in the form of a cone to maintain the expansion.

### Postoperative management

Ofloxacin-containing eye drops were prescribed four times per day for 7 days in the RSHSI group, and the silicone intubation tubes were left in place for 6 months. After extubation, nasolacrimal canal lavage was applied to all patients on removal of the tube, and then again every 15 days over the course of at least 3 months. For patients in the En-DCR group, topical steroids given over a period of 6 months. Patients were followed up at their first day and first week, and then after 1, 3, 6 and 12 months for endoscopic removal of crusts around the lacrimal window and for lacrimal passage irrigation.

### Follow-up

All patients in the RSHSI group were followed up after their first day and first week, and then after 1, 3, and 6 months with the tube. After 6 months, the tube was removed and lacrimal passage irrigation was immediately performed; it was subsequently performed once every 15 days for a period of at least 3 months. Six months after extubation, we finally evaluated the therapeutic effects according to patient satisfaction and lacrimal passage irrigation outcome. At the same time, we recorded patient satisfaction levels, indexed by VAS scores. With regard to the En-DCR group, the patients were also followed up under endoscopy after their first day and first week, and then after 1, 3, and 6 months, and 1 year. During follow-up, we recorded VAS scores and evaluated the success rate at the last visit, as noted above.

### Primary and secondary outcomes

The main outcomes of this study were assessed by the success rate and evaluation of treatment effectiveness: at the final visit, improvements in epiphora were recorded to index the success rate. Table 1 shows the evaluation standard, based on clinical symptoms and lacrimal passage irrigation outcome. The secondary outcome was cost-effectiveness including intraoperative visual analogue scale, bleeding volume, operating time, duration and total cost of hospitalisation and postoperative epiphora VAS scores.

**Table 1:** The Curative effect Appraisement.

Curative effect evaluation	Epiphora and Lacrimal Passage Irrigation
Cure	postoperative epiphora disappeared
	no reflux after lacrimal passage irrigation
Effective	clinical symptom remission
	a little reflux after lacrimal passage irrigation
Invalid	no effect on epiphora
	a lot reflux after lacrimal passage irrigation

### Intraoperative visual analogue scale

Pain, as an index of patient discomfort, was graded from 0 (*none*) to 10 (*worst pain*) following the operation, to assess the degree of trauma. The VAS scores of the RSHSI patients were recorded immediately after the operation, since it was performed under local anaesthesia, but those in the En-DCR group were recorded at 6 h after the operation, by which time the patients had recovered from the effects of the general anaesthetic.

### Bleeding volume

The bleeding volume was recorded for each operative eye in both groups to evaluate degree of trauma and the complexity of the procedure.

### Operating time

For each operative eye, the time taken to complete surgery in the RSHSI group was given by the time between dilatation and unobstructed lacrimal irrigation. In the En-DCR group, the operative time was the time between flap incision and packing.

### Duration and total cost of hospitalisation

For each NLDO patient, the socioeconomic benefits of the two types of surgery were compared.

### Postoperative epiphora VAS scores

Visual analogue scale scores were used to assess improvements in epiphora based on degree of patient satisfaction.

### Statistical analysis

Data are given as means ± standard error, or as frequencies. To test the homogeneity of the two study groups, a chi-squared test was performed to compare the sex and operative eye ratios, and a two-tailed independent t-test was used to compare age. A two-tailed independent t-test was also used to compare the cost-effectiveness of the two types of operation, as indexed by VAS scores, operating time, bleeding volume, hospitalisation time, and total cost. All statistical analyses were performed using the SPSS statistical software package (IBM Corp., Armonk, NY, USA). For all comparisons, a P-value < 0.05 was considered to indicate statistical significance.

### Ethical approval

This trial was approved by the Research Ethics Committee of the Institute of Basic Research in Clinical Medicine, Third Affiliated Hospital of Sun Yat-sen University. All patients were asked to provide written, informed consent before participating.

### Transparency

Hy Deng affirms that this manuscript is an honest, accurate, and transparent account of the study; that no important aspects of the study have been omitted; and that any deviations from the study protocol have been explained.

## Results

### Enrolment

**Figure 6:**
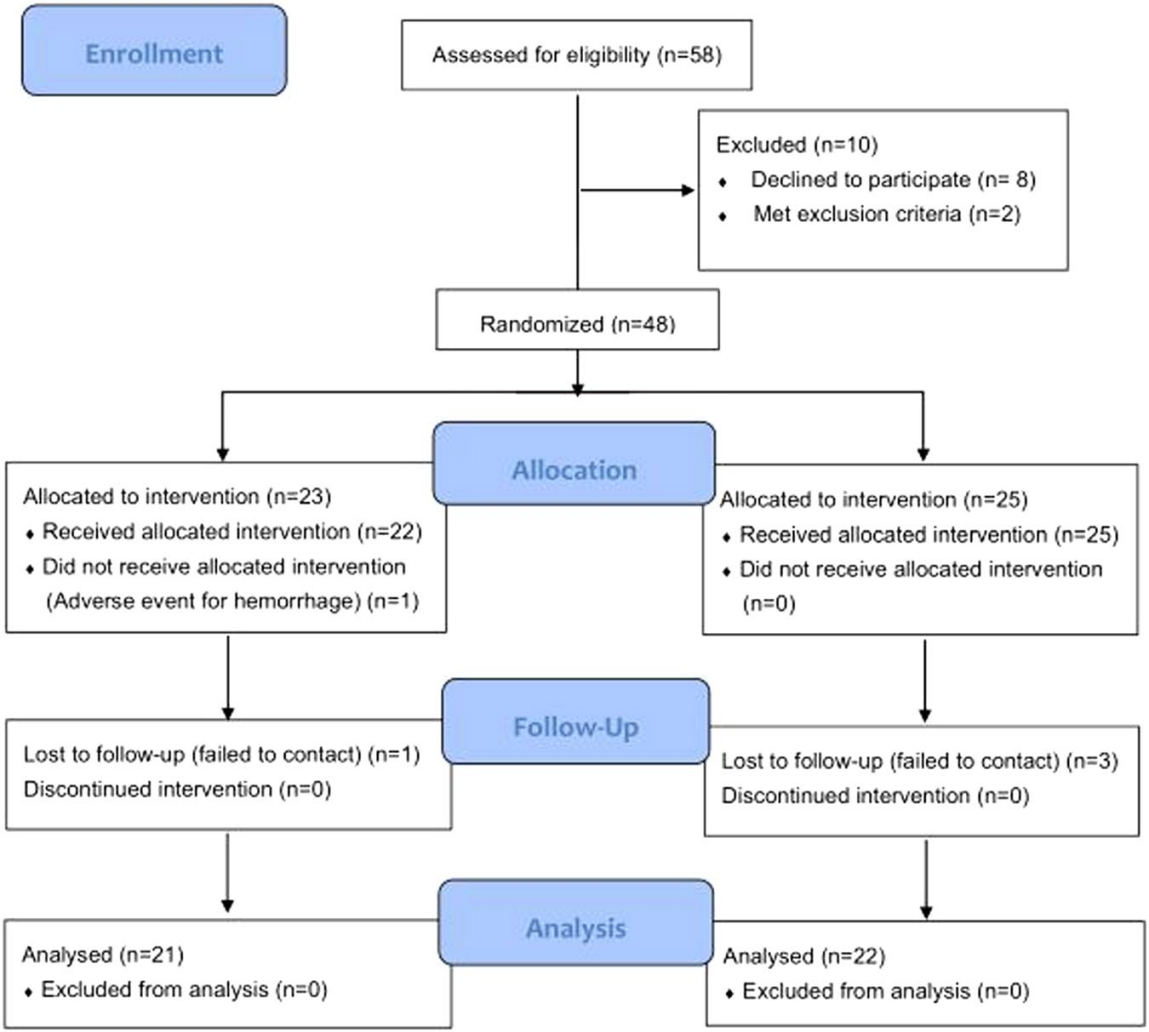
CONSORT 2010 Flow Diagram^19^.

In total, 58 patients were initially deemed suitable for inclusion in our study (Fig. 6). Among them, eight patients refused to participate and two met the exclusion criteria. After the study began, an adverse event occurred in the RSHSI group and four of these patients were lost to follow-up. Finally, a total of 43 patients (n = 21 in the RSHSI group and n = 22 in the En-DCR group) were enrolled for analysis.

### Characteristics of the population

Forty-three NLDO patients (50 eyes) recruited from the Department of Otorhinolaryngology and the Department of Ophthalmology were divided into two groups; there were no significant differences between the groups (Table 2). Twenty-five eyes (of 5 males and 18 females aged between 21 and 83 years; mean age = 47.53 ± 17.35 years) underwent surgery in our clinic using the novel RSHSI procedure performed under nasal endoscopy. Of these 25 eyes, 10 were right eyes and 15 were left eyes, of 17 monocular and 4 binocular patients. The duration of NLDO ranged between 1 and 23 years, with a mean duration of 6.06 ± 5.47 years. Twenty-five eyes (of 5 males and 17 females aged between 25 and 65 years; mean age = 48.50 ± 10.64 years) underwent the traditional En-DCR surgery. Of these 25 eyes, 15 were right eyes and 10 were left eyes, of 19 monocular and 3 binocular patients. The duration of NLDO in this group ranged between 6 months and 10 years, with a mean duration of 4.48 ± 5.83 years. However, before the study was concluded, an adverse event, persistent epistaxis, occurred after RSHSI; this was initially suspected of being nasal submucosa pseudocanal, but the haemorrhage stopped after removal of the tube and the affected patients were removed from the study.

**Table 2:** Baseline Characteristics of patients in the Analysis.

Characteristics at Baseline	RSHSI (n_1_ = 25, n_2_ = 21)	En-DCR (n_1_ = 25, n_2_ = 22)	P value	t or χ^2^	95% for CI
*Age (yrs)	47.53 ± 17.35	48.50 ± 10.64	0.863	−0.174	(−12.406, 10.458)
*History (yrs)	6.06 ± 5.47	4.48 ± 5.83	0.458	0.753	(−2.710, 5.863)
^#^Female:male	18:3	17:5	0.477	0.102	(0.117, 2.744)
^#^Right:left	10:15	15:10	0.157	2.000	(0.143, 1.378)

Note: *Two-tailed independent t test. ^#^Two-tailed chi-square test. Abbreviations: RSHSI, Recessive Spherical Headed Silicone Intubation. En-DCR, Endonasal Dacryocystorhinostomy. CI, Confidence Interval. n_1_ = the number of operative eyes in each group. Right: left was analysed based on n_1_. n_2_ = the number of NLDO patients in each group. Age, history and gender were analysed based on n_2_.

### Epiphora improvement

**Figure 7:**
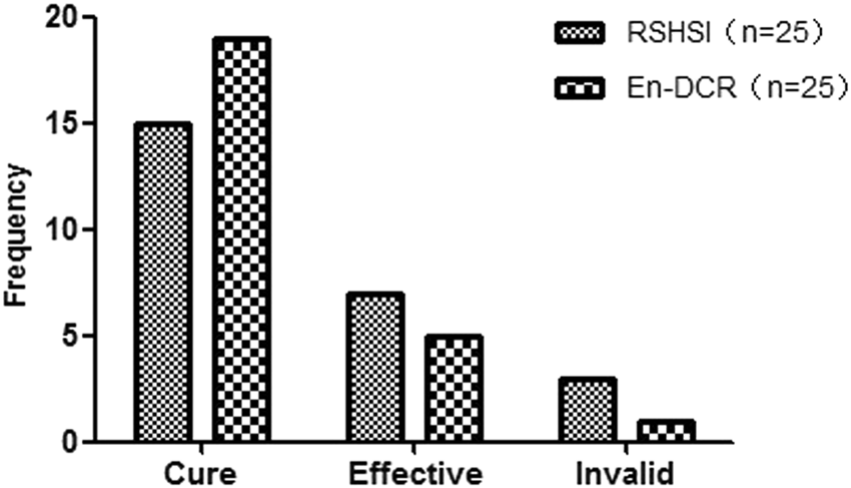
Outcomes of epiphora improvement respectively after the RSHSI or En-DCR described as frequency. n = the number of operative eyes. (χ^*2*^ = 1.804, P = 0.406).

The evaluation criteria for epiphora improvement are given in Table 1. Among the 25 eyes in the RSHSI group, 15 cases (60.00%) were classified as cured, 7 cases (28.00%) were effective, and 3 cases (12.00%) were invalid. The success rate was 88.00%. Correspondingly, in the En-DCR group, 19 cases (76.00%) were cured, 5 cases (20.00%) were effective, and 1 case (4.00%) was invalid, with a 96.00% success rate. There was no significant difference in success rate between the two groups (Fig. 7).

### Postoperative cost-effectiveness analysis

**Figure 8:**
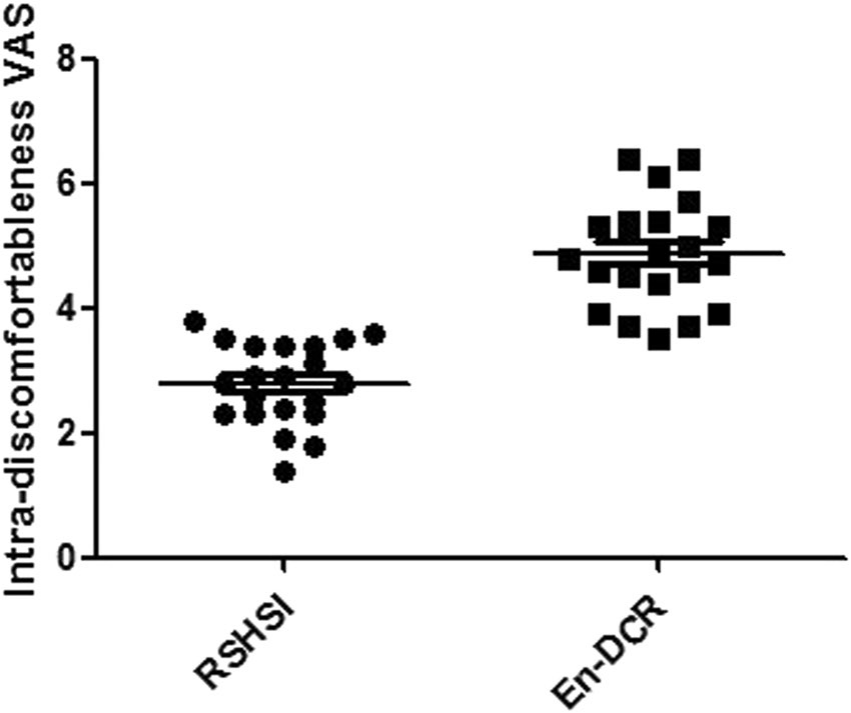
Comparison of the intra-operative discomfort VAS scores for each patient after the operation. (n = NLDO patients, respectively n_1_ = 21, n_2_ = 22, P = 0.000).

**Figure 9:**
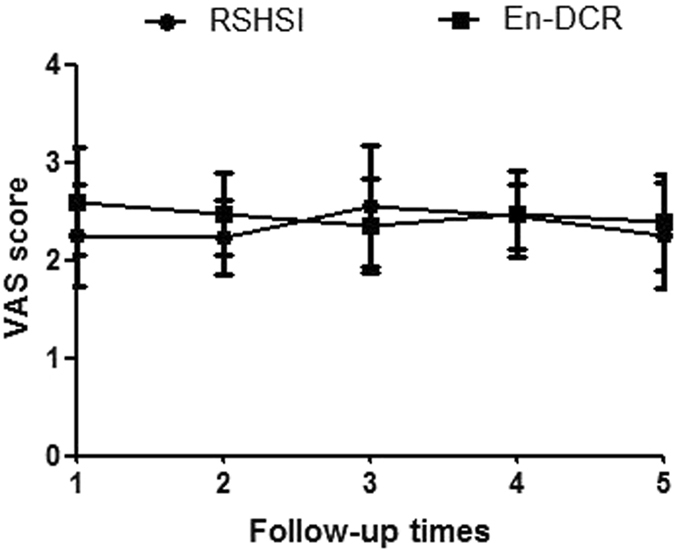
Postoperative epiphora improvement five times in one year follow-up including 6 months tube placement and 6 months after extubation. (n = operative eyes, P > 0.050).

**Figure 10:**
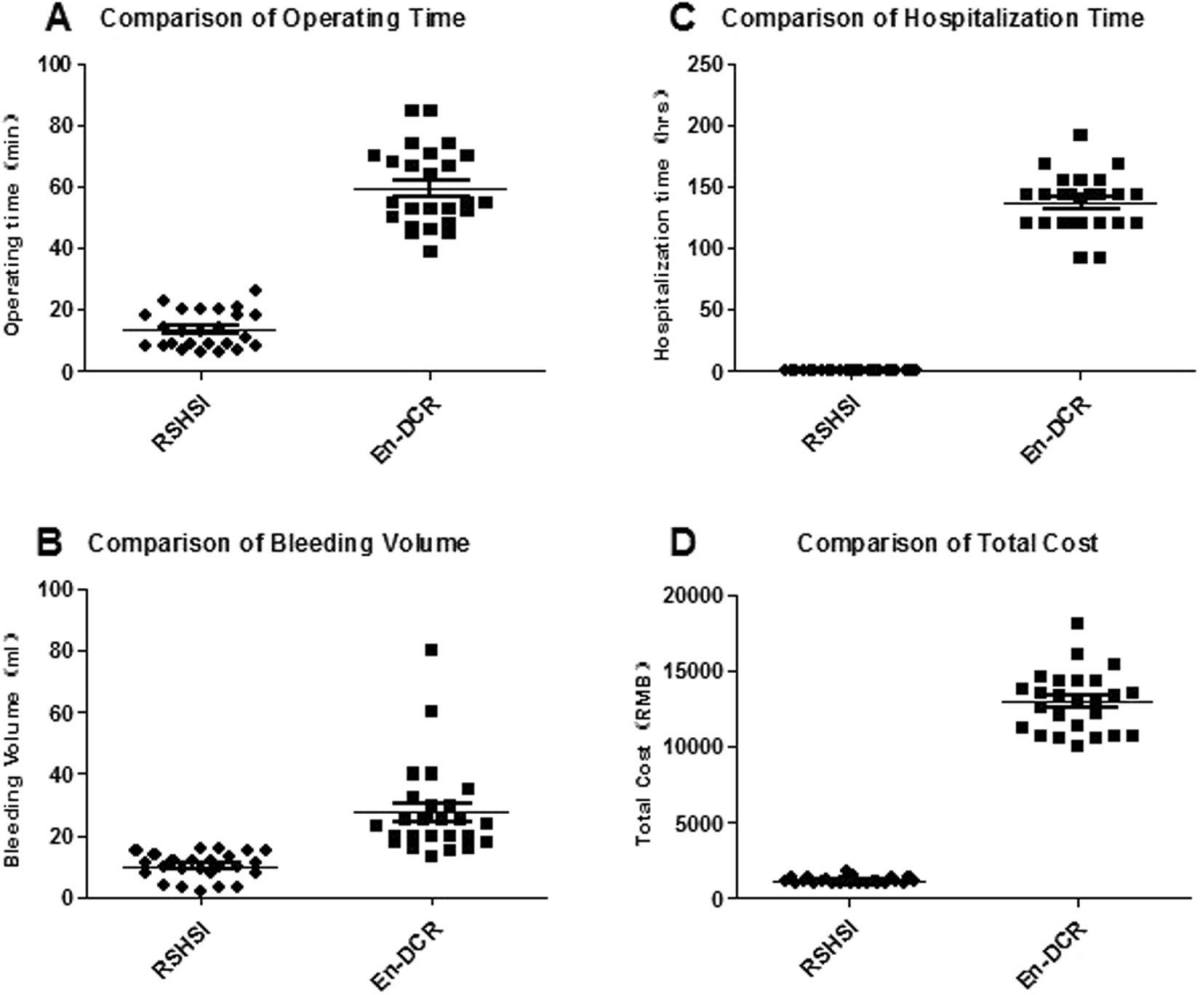
Comparison of the postoperative cost-effectiveness between the two groups. (**A**) Operating time in minutes for each operative eye (n = operative eyes, n = 25, P = 0.000). (**B**) Bleeding volume in ml for each operative eye (n = operative eyes, n = 25, P = 0.000). (**C**) The length of hospital stay for every NLDO patient described as hours (n = NLDO patients, n_1_ = 21, n_2_ = 22, P = 0.000). (**D**) The payment of each NLDO patient after RSHSI or En-DCR (n = NLDO patients, n_1_ = 21, n_2_ = 22, P = 0.000).

To assess the follow-up social-economic benefits, we compared intraoperative VAS and multiple epiphora VAS scores (Table 3, Fig. 8), operating time, bleeding volume, hospitalization time, and total cost. After a two-tailed independent t test, we found that there was no significant difference in multiple epiphora VAS scores between the two groups (Table 4, Fig. 9), but there were significant differences in the other factors (Table 5, Fig. 10).

**Table 3:** Comparison of intra-operative discomfortableness VAS scores.

Visual Analogue Scale	RSHSI (n = 21)	En-DCR (n = 22)	P value	95% for CI
Intra-discomfort	2.84 ± 0.64	4.88 ± 0.84	0.000	(−2.518, −1.576)

Two-tailed independent t test. Abbreviations: CI, Confidence Interval. n = the number of NLDO patients.

**Table 4:** Comparison of postoperative epiphora VAS scores.

Follow-up Times	RSHSI (n = 25)	En-DCR (n = 25)	P value	95% for CI
1 week	2.36 ± 0.80	2.60 ± 0.55	0.222	(−0.630, 0.150)
1 month	2.32 ± 0.59	2.45 ± 0.42	0.340	(−0.432, 0.152)
3 months	2.67 ± 0.76	2.40 ± 0.48	0.136	(−0.089, 0.633)
6 months	2.55 ± 0.59	2.48 ± 0.43	0.606	(−0.219, 0.371)
1 year	2.35 ± 0.64	2.43 ± 0.44	0.592	(−0.397, 0.229)

Two-tailed independent t test. Abbreviations: VAS, Visual Analogue Scale; CI, Confidence Interval; n, the number of operative eye. (Note: The 1 year follow-up in our study was composed of 6 months tube placement and 6 months follow-up after extubation).

**Table 5:** Comparison of cost-effectiveness between the two kinds of operation.

Cost-effectiveness	RSHSI (*n = 25, ^#^n = 21)	En-DCR (*n = 25, ^#^n = 22)	P value	95% for CI
Operating time (min)	13.40 ± 6.007	59.64 ± 12.786	0.000	(−51.921, −40.559)
Bleeding volume (ml)	4.96 ± 1.989	27.60 ± 14.950	0.000	(−28.854, −16.426)
Hospitalization time (hrs)	0.50 ± 0.00	137.280 ± 23.085	0.000	(−146.3094, −127.2506)
Total cost (RMB)	1194.24 ± 39.533	11663.75 ± 1384.383	0.000	(−11749.523, −9188.568)

Two-tailed independent t test. CI, Confidence Interval. *n, the number of operative eyes in each group. ^#^n, the number of NLDO patients in each group. Operating time and bleeding volume was analysed based on *n. Hospitalization time and total cost was analysed based on ^#^n.

### Safety

In both groups, the severity of most of the adverse events (AEs) was mild or moderate. There was no significant difference in the AE rate between groups.

## Discussion

Symptomatic nasolacrimal obstruction is a common, multifactorial clinical condition that has a major impact on the quality of life and can result in many complications^15^. It typically arises in middle-aged females because of their long and narrow nasolacrimal ducts. The incidence rate in females in our study was 84.0%, which is in agreement with the literature^1^. The most common type of surgery for NLDO is En-DCR, which can relieve the nasolacrimal duct ligation and reconstruct the drainage channel, but has disadvantages including nasal wounds, high cost, and a destructive effect on the lacrimal pump. In our study, RSHSI was able to avoid all of these problems above. However, blind intubation has traditionally been ineffective because of the common occurrence of false localisation of the nasolacrimal duct. Traditional surgery, which always relies on experience or personal feelings, is time-consuming, and fail to hook the line, because of the operation being performed blind, results in mucosal injury that in turn leads to scar formation, nasal cavity adhesion, poor clinical outcome, and various complications. Moreover, junior doctors are unable to acquire expertise in this traditional surgery in a step-by-step manner from experienced surgeons, so their learning curve is protracted. In our study (Fig. 4), the images provided by nasolacrimal system endoscopy, which allows direct visualisation of the area to be treated, enabled the lacrimal punctum to the opening of the nasolacrimal canal to be visualised^16^. This allowed the nasal submucosa pseudocanal to be avoided, the nasal mucosa to be protected, and nasal bleeding to be prevented^12, 14, 17^. In addition, RSHSI performed under endoscopic guidance can identify nasal diseases, and any negative effects from the procedure lessen over time^12, 13^. Silica gel is currently the most common material used in NLDO surgery; it is non-toxic and non-irritating, with good histocompatibility, and can endure long-term catheterisation. What’s more, our silicone head has a spherical-cavity structure with an outer diameter of 4.2 mm, which can be relatively stably fixed on the lacrimal sac and also remarkedly reduce the incidence of lacrimal-sac obstruction and junction bostruction between the nasolacrimal duct and the lacrimal sac^16^. Furthermore, the silicone tube can be re-implanted, or the operation can be converted to En-DCR if RSHSI failure occurs. Above all, RSHSI under nasal endoscopy is suitable for intraocular surgery and patients unsuitable for En-DCR due to poor physical condition. RSHSI requires no general anaesthesia and has many advantages, such as short operative duration and rates of reduced injury and bleeding, and conferring a parallel curative effect to that of En-DCR^5, 18^. Moreover, patients can live without restriction with the tube in place for a long period. However, there was no significant difference in surgical outcomes between the two groups in our study, indicating that both RSHSI and En-DCR can effectively treat NLDO, and show similar outcomes.

Our data show that the VAS scores of the RSHSI group were clearly lower than those of the En-DCR group (Table 3 and Fig. 8), indicating that RSHSI is minimally invasive, less time-consuming, well-tolerated, and satisfactory for use on an outpatient basis. However, patients in the En-DCR group may have been uncomfortable due to feelings of “stuffiness” and body aches after general anaesthesia. Nevertheless, in our analysis of patient satisfaction in terms of epiphora improvement, we found that, in the RSHSI group, satisfaction was initially high, and then decreased; peak satisfaction appeared at month 3 of tube placement. This shows that the obstruction in the nasolacrimal duct became less noticeable after intubation, such that patients felt comfortable; then, in line with adaptation by the silicone tube, patients felt better (classified as satisfactory based on lower VAS scores). Unfortunately, due to a lack of attention from patients to good eye health, or the presence of upper respiratory infection or mild displacement, purulent tear and discomfort are likely to be present after several months, leading to higher VAS scores. Despite this, there was no significant difference in terms of the operation success rate between the two groups. At month 6 after tube placement, patients felt well because they had adapted better to the tube, and also due to removal of the obstruction in the lacrimal duct; therefore, they felt unrestricted by the tube and VAS scores decreased accordingly. During the 6 month period after extubation, we evaluated the operation success rate according to patient satisfaction, as indexed by VAS scores, and lacrimal passage irrigation. The VAS scores among the En-DCR group showed a slight downward trend, implying that the high initial VAS score was related to operative wounds and nasal packing, although by that stage clearance of the obstruction should have already provided relief. As the swelling reduced, stuffiness lessened and the purulent secretion diminished; thus, VAS scores were also reduced. With regard to short-term effects after extubation, the VAS scores of the RSHSI group were lower than those of the En-DCR group, indicating that RSHSI was more effective than En-DCR (Table 4 and Fig. 9). Overall, the short-term curative effect of RSHSI was superior to that of En-DCR, but effectiveness may decline slightly due to re-obstruction or tube displacement. However, the curative effect should improve with lacrimal passage irrigation. RSHSI leaves no facial scar and the physiological lacrimal pump mechanism is preserved. Moreover, a shorter operation time is required for this procedure, the medial canthal ligament remains intact and patients can be ambulatory at an early stage. RSHSI results in minimal blood loss and, above all, shows good effectiveness. Our results indicate that the two different kinds of surgery compared herein are both acceptable for treating NLDO. RSHSI, however, provides better outcomes during short-term follow-up, especially for emergency cases.

Our study showed that RSHSI and En-DCR have a similar therapeutic effect (Fig. 7); combining the rhinological approach of endoscopic sinus surgery with the ophthalmic approach of retrograde tube intubation can greatly reduce the degree of operative difficulty, operating time, and nasal mucosa injury. The procedure for RSHSI under nasal endoscopy is classified as low-risk, is well-tolerated, and can be performed on an outpatient basis, thereby lowering the cost in terms of both time and money (Table 4 and Fig. 10). To summarise, RSHSI is a practical and cost-effective choice for NLDO patients. Moreover, it serves to optimise the basic skills of surgeons and provides better quality of life for patients. In our opinion, RSHSI merits wider clinical application, especially in general hospitals. As noted by Alanon-Fernandez *et al*.^14^, “To maximise the success rate of surgery in the simplest way”, is our long-term goal, and the study of RSHSI has therefore been prioritised.
